# Muscle-tendon weakness contributes to chronic fatigue syndrome in Gaucher’s disease

**DOI:** 10.1186/s13018-019-1452-y

**Published:** 2019-11-21

**Authors:** Mercedes Roca-Espiau, Marcio Andrade-Campos, Jorge J. Cebolla, Laura López de Frutos, Blanca Medrano-Engay, Maria-Pilar López-Royo, Pilar Giraldo

**Affiliations:** 1 Fundación Española Estudio y Tratamiento de la Enfermedad de Gaucher y Otras Lisosomales (FEETEG), Zaragoza, Spain; 2 Radiologic Centre CEMEDI, Zaragoza, Spain; 3 Grupo de Investigación en Enfermedad de Gaucher (GIIS-012), Unidad de Investigación Traslacional, Fundación Instituto de Investigación Sanitaria Aragón, Zaragoza, Spain; 4 Departamento de Hematología, Hospital del Mar, Barcelona, Spain; 5 Departamento de Bioquímica, Biología Molecular y Celular, Facultad de Ciencias, Universidad de Zaragoza, Zaragoza, Spain; 6 iPhysio Group, Universidad San Jorge, Zaragoza, Spain

**Keywords:** Gaucher disease, Fatigue, Strain-elastography, Achilles tendon stiffness, QoL

## Abstract

**Background:**

Chronic fatigue (CFg) is a prevalent symptom in Gaucher disease (GD) at diagnosis (79%) and remains in a quarter of patients after years of therapy. Bone abnormalities are present in over 70% and peripheral neuropathy in about 11% of the patients, which contributes to the disabling and debilitating complications. Our hypothesis is that other factors such as muscle-tendinous weakness could have influence in the development of CFg.

**Methods:**

We have evaluated the fiber structure and elasticity of muscle-tendinous unit by strain-elastography (S-ELA) and analyzed their influence in the CFg. S-ELA study was performed in Achilles tendon in 25 type 1 and two type 3 GD patients, all of them with fatigue and were on enzymatic replacement therapy for mean 13 years; simultaneously, bone marrow burden by MRI and calcaneus ultrasound densitometry were evaluated. Blood cell counts, plasma biomarkers, *GBA1* genotyping, and SF36 quality of life scale (QoL) were also performed. Statistical analysis: descriptive and comparative test.

**Results:**

All patients showed a normal Achilles tendinous structure. Abnormal stiff grade 2–3 was found in 17/27 (62.9%); in 11/27 (40.7%) of patients, the alteration was bilateral. There were no correlations between the S-ELA results to other variables; nevertheless, a significant correlation between the degree of tendon hardness and the low score on the QoL scales (*p* = 0.0035) was found. The S-ELA is a sensitive painless, fast, and low cost method to detect muscle-tendinous subclinical dysfunction that could contribute to CFg in GD. The identification of subclinical tendon alteration would be a sign of alarm, focused on the risk of development of bone complications.

**Conclusion:**

Intratendinous alteration in strain-elastography is an independent variable in GD patients with persistent fatigue.

## Background

Chronic fatigue (CFg) is the most frequent symptom of patients suffering from Gaucher disease (GD) (79% [1]); the use of enzymatic replacement therapy (ERT) has changed the natural history of the disease; its long-term use leads to an improvement of visceral, hematological, and bone symptomatology; and the majority of type1 (GD1) patients achieves the goals of therapy. However, in several patients on long-term ERT, the CFg is still present in almost one-quarter of them (24.5%) [1, 2].

There is limited information about the cause of CFg, although it is mainly attributed to hypermetabolism, myopathy, or an increase in different plasma cytokines concentrations [2, 3]. Nevertheless, there are several factors such as anemia, pain, depression, anxiety, sleep disturbances, emotional distress, physical activity, or medication side effects that could be involved [2, 3]). Bone disease is present in over 70% of the GD patients; in addition, peripheral neuropathy has been described in about 11% of them, which contributes to the disabling and debilitating complications in GD1 [4, 5]. However, other factors including muscle-tendinous weakness could have influence in the development of fatigue. The prevention of bone-related adverse events such as osteoporosis, pathological fractures, deformation, and osteonecrosis remains as a clinically meaningful need that has not been solved yet and which has an important impact on the quality of life (QoL) [6, 7]. The use of long-term ERT decrease the incidence of intraosseous vascular complications; however, to date, there are no good prognostic markers to identified patients at high risk and even patients on ERT can develop bone complications.

Our hypothesis is that other factors including muscle-tendinous weakness could have influence on the development of fatigue. Only a few studies have evaluated the fatigue in GD, and objective quantification has been performed in the previously mentioned studies ([3].

The aim of this study is to evaluate the myotendinous unit in a group of adult GD patients with CFg, by a simple, non-invasive new technology based on strain-elastography (S-ELA) technique which allows the assessment of the biomechanical and structural properties of tissues by measuring their stiffness. A secondary objective is to study the correlation of the results of the S-ELA study with the main characteristic parameters related to bone disease.

## Patients and methods

### Design and studied population

This study was designed as a cross-sectional case-control, in 25 adults suffering from GD1 and 2 GD type 3 patients, diagnosed and follow-up periodically in our Expert Unit on Lysosomal diseases. All patients were diagnosed by enzymatic and genotyping studies; also, they were evaluated and followed according to the Spanish Guidelines [8] which include clinical, biological, hematological, and biomarkers evaluation every 6 months and bone marrow burden study by magnetic resonance imaging (MRI), control of visceral size by abdominal MRI, and ultrasound bone densitometry every year.

A total of 12 females and 15 males were included (mean age = 41.0 years old, 18.0–62.0 years old). Six patients were splenectomized before the ERT. All of them reported symptomatic CFg despite of several years under treatment (mean 13.0 years, 2.0–25.0 years).

The control group was composed by 20 subjects, with a gender ratio 1/1 and a mean age of 45.0 years old; 18.0–59.0 years old).

In all patients, a clinical interview and physical examination have been performed, taking special attention to the presence of neurological manifestations, therapy of GD, previous history of diabetes, associated endocrine disorders, other comorbidities, or concomitant treatments with steroids. A control group of 20 healthy subjects matched by gender and age were also studied.

All individuals and controls provided written informed consent and the Aragon regional government’s Ethics Committee for Clinical Research, (Zaragoza, Spain) approved the study. The research was conducted in accordance with the principles stated in the Declaration of Helsinki–Ethical Principles for Medical Research Involving Human Subjects, Helsinki, Finland, 1964 and as amended in Fortaleza, Brazil, 2013.

### Strain-elastography exam

All patients underwent an exam of both Achilles tendons with real-time tissue S-ELA procedure (Hitachi system EUB-8500, with L54 M transducer, frequency 6–13 MHz) and gray-scale sonographic findings performed simultaneously (B mode) [9, 10].

**Fig. 1:**
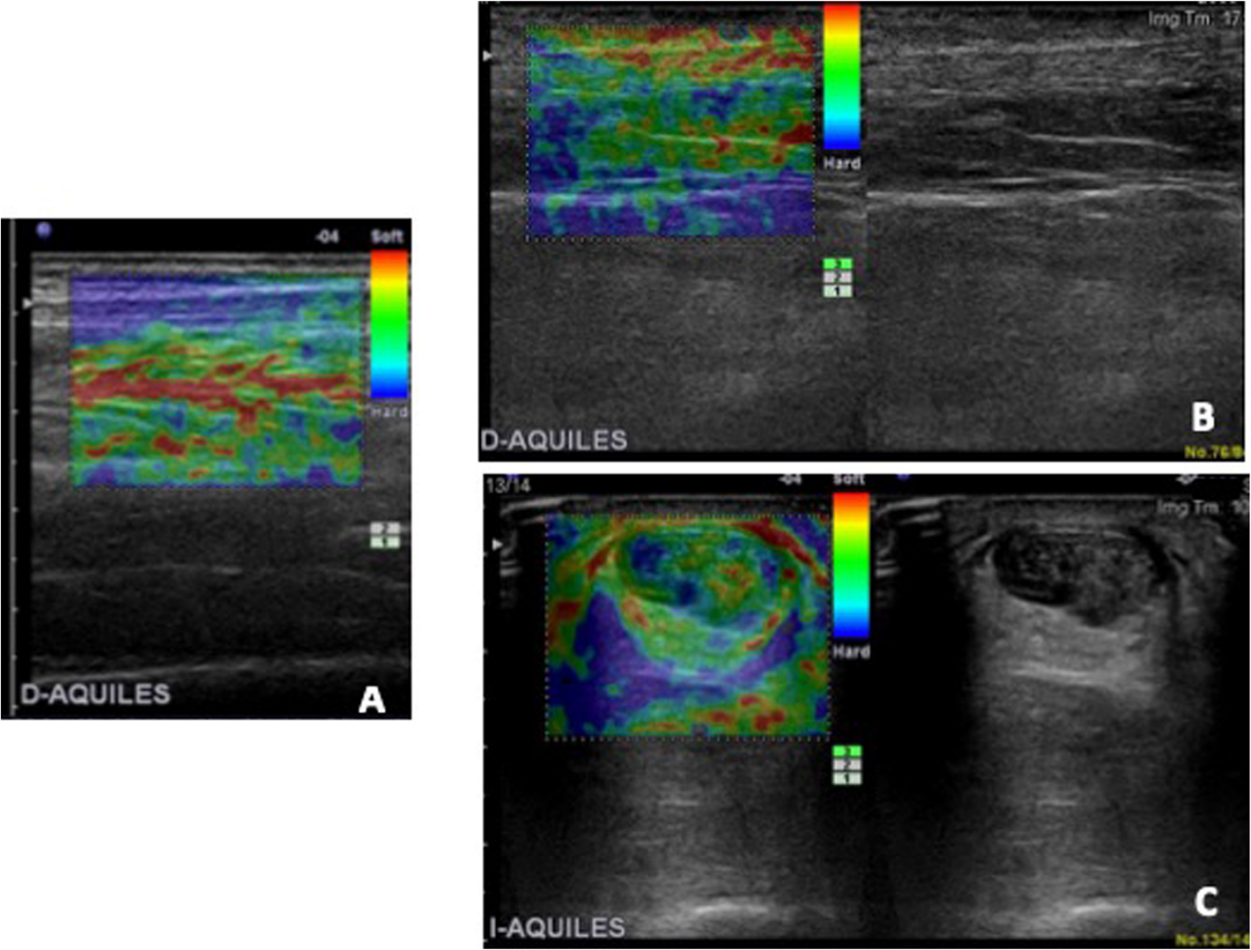
Strain-Elastography. **a** Normal stiffness. **b** Abnormal elasticity greater deformability. Red color. **c** Morphological and stiffness alteration

Longitudinal and axial scans in three areas of Achilles tendon (in the proximal area near the myotendinous junction, in the middle, and in the distal area of the insertion of the tendon in the calcaneus bone) enabled the assemblage of several integrated images to evaluate its morphological structure, as well as its stiffness and to classify it using a semi-quantitative score of different colors to represent stiff tissue (blue, grade 1) to more soft tissue (green-yellow, grade 2) and (yellow-red, grade 3) [9] (see Fig. 1).

### Bone assessment

The bone marrow was evaluated by MRI following the Spanish MRI score (S-MRI) protocol [11] and bone mineral density (BMD) by the mechanical energy transmitted by the bone expressed in broad ultrasound attenuation (BUA) [12]. According to reference tables, the values then have converted to a T-score or a Z-score.

### Visceral volume

Abdominal MRI to determine liver and spleen volume have been performed at the same time.

### Blood examination

Blood cell counts, basic biochemical determinations including iron and ferritin levels, liver enzymes, lipid profile, and plasma biomarkers related to GD were performed.

Among GD plasma biomarkers, it were analyzed the chitotriosidase activity (ChT) using 4-methylumbelliferyl-β-d-*N*,*N*′,*N*″-triacetylchitotrioside at non-saturating concentrations to avoid substrate inhibition [13]. The concentrations of chemokine (C-C motif) ligand 18/pulmonary and activation-regulated chemokine (CCL18/PARC) through ELISA technique [14] and glucosylsphingosine (Lyso-Gb1) were determined by a non-laborious method using chromatography-grade methanol and d-glucosyl-β-1,1′-d-erythro-sphingosine-d5 as internal standard, then separated by liquid chromatography system and detected and quantified by coupled mass spectrometry equipment [15].

### Quality of life evaluation

The application of the Spanish version of the Health Survey SF-36 questionnaire [16] was performed at the time of the S-ELA. The questionnaire contains 36 items divided in eight health domains: physical function, role limitation due to physical problems, bodily pain, general health, vitality, social functioning, role limitation due to emotional problems, and mental health. The eight domains can be used to provide physical and mental component summary scores. Each category was scored on a scale of 0–100 were 0 represent the worst overall health status and 100 the best health status. To determine the variability in the questionnaire scores, we have compared the results obtained in the patients with the values published for the Spanish general population [16].

### Statistical analyses

Statistical analyses were performed using SPSS (SPSS version 21.0, IBM®, USA) software. Qualitative variables were given as frequencies and percentages and compared using Chi-squared test. Quantitative variables were analyzed for normal distributions by the Kolmogorov-Smirnov test. Normally distributed variables were given as mean and standard deviation and correlated using Pearson test.

All statistical tests considered and took as bilateral significance a level of α = 0.05.

## Results

### Demographical characteristics

The general characteristics are detailed in Additional file 1: Table S1. All the patients achieved for the long-term hematological and visceral goals of therapy; none of them registered anemia or iron lack, and no bone crisis has been registered in the 2 years previous. Concomitant chronic diseases were described in 13 patients (48.1%).

### Strain-elastography assessment

The tendons in the 20 healthy subjects show a blue color (grade 1), consistent with normal stiff tendon tissue. Both Achilles tendons were examined (54) in all the 27 patients; alterations in stiffness were observed in 17 (62.9%) patients, (registered as intratendinous color alterations). The gradation according to S-ELA method was grade 3: 12/54, grade 2: 16/54, and grade 1: 26/54. Globally, 28 tendons showed alterations in the S-ELA study, 11 (40.7%) patients showed both tendons affected, six (22.2%) only one, and ten (37.1%) showed no alterations at all. The ultrasound gray scale showed no changes in the fibrillar pattern, tendon thickness, or echo-structure. The detailed data are shown in Table 1.

**Table 1:** General data

Patient	S-MRI	R-S-ELA grade	L-S-ELA grade	T-score	Z-score	ChT nmol/mL/h NR: 4.0–133.0	CCL18/PARC ng/mL NR: 12.0–165.0	Lyso Gb1 ng/mL NR: < 0.88	Hb g/dL	Ferritin ng/mL NR: 15–250
011	12	1	1	− 0.60	0.09	126	62	1.56	12.8	201
02	2	1	1	1.51	1.37	63	55	2.50	13.8	353
03	5	2	2	− 0.42	− 0.42	50	73	< 0.88	17.3	300
04	9	3	3	− 0.59	− 0.27	6575	553	39.57	15.5	114
05	11	3	2	− 1.95	− 1.66	1697	360	40.95	13.8	2000
06	2	1	1	− 0.80	0.39	96	76	1.50	14.0	93
07	0	2	2	1.07	1.45	377	99	3.10	13.8	553
08	0	2	1	− 0.61	− 0.71	394	86	6.50	13.8	353
09	15	3	1	− 2.43	− 1.47	208	150	50.32	14.1	29
10	6	1	2	− 2.50	− 1.8	5136	529	131.25	12.6	385
11	6	3	3	− 2.50	− 1.7	351	233	4.20	12.8	60
12	8	2	2	0.42	0.61	1368	239	15.10	15.5	360
13	4	1	1	− 2.64	− 1.59	1840	328	21.90	13.5	220
14	18	3	3	− 2.06	− 2.32	–	265	2.73	13.8	250
15	8	3	2	− 1.40	− 1.43	974	265	2.80	13.5	74
16	8	3	1	1.12	1.15	1849	474	57.90	13.3	700
17	4	1	1	− 1.49	− 0.59	337	76	5.10	12.2	65
18	11	2	1	− 0.60	− 1.54	2972	925	144.0	14.1	200
19	0	1	1	− 0.98	0.31	46	31	< 0.88	13.8	50
20	0	1	1	0.24	− 0.15	809	88	9.90	15.7	74
21	7	3	2	1.27	0.85	5740	526	124.6	13.0	
22	12	1	1	− 2.29	− 1.96	1700	293	60.10	14.0	30
23	8	1	1	0.56	0.56	90	60	3.5	13.3	91
24	12	1	1	− 1.56	− 2.24	491	201	13.4	14.7	27
25	7	2	2	0.74	3.13	541	232	4.5	13.6	1733
26	13	2	1	0.76	2.2	1554	776	124.5	14.4	1349
27	4	1	1	− 0.21	− 0.68	108	53	< 0.88	13.5	350
Total	7.1 0–18			− 0.67 1.51; − 2.39		1480 50–6575	253 31–776	23.3 < 0.88–144		399.3 27–2000

*S*-*MRI* Spanish MRI score, *R*-*S*-*ELA* Achilles right strain-elastography, *L*-*S*-*ELA* Achilles left strain-elastography, *ChT* Chitotriosidase

The Pearson test does not show any correlation between the S-ELA grade and other variables, with age (*p* = .134), with the S-MRI (*p* = .025), T-score (*p* = .620), biomarkers: ChT (*p* = .423), CCL18/PARC (*p* = .049), Lyso Gb1 (*p* = .275), Ferritin (*p* = .151), or time on therapy (*p* = .174), only with the related physical items and general health in the QoL evaluation (*p* = .003). Therefore, the S-ELA is an independent variable in GD patients with CFg with a clear impact on the QoL status.

### Bone evaluation

The mean S-MRI was 7.1 points (range 0.0–18.0). T-score: mean − 0.67 (range − 2.68–1.51). Advanced bone disease was present in ten patients (37.0%) who showed several vascular complications in the MRI. The osteopenia (T-score − 1 to − 2.5) was registered in eight patients (34.7%).

### Analytical data

The genotype distribution was NP_000148:p.Asn409Ser + p.Leu483Pro 59.2%, p.Asn409Ser + other 37.0%, p.Leu483Pro + other 11.1%, other + other 3.7% (see Additional file 1: Table S1).

The mean hemoglobin concentration in males was 14.5 g/dL (range 13.0–17.3) and in females was 13.3 g/dL (range 12.2–14.1). The biochemical inflammatory biomarkers were higher than laboratory cut-off: Ferritin: mean = 399.3 ng/mL (range 27.0–2000.0); ChT: mean = 1480 nmol/mL/h (range 46–6575); CCL18/PARC; mean = 253.1 ng/mL (range 53.0–925.0); Lyso-Gb1: mean = 23.3 ng/mL (range 0.0–144.0) (see Table 1).

### Quality of life assessment

A lower score in all dimensions of the QoL in GD patients compared with general population was observed. The QoL results show the lower punctuation in physical role, bodily pain, emotional role, and general health. A significant correlation (*p* = 0.003) has been observed between the grade of the S-ELA and the general health QoL scale.

**Fig. 2:**
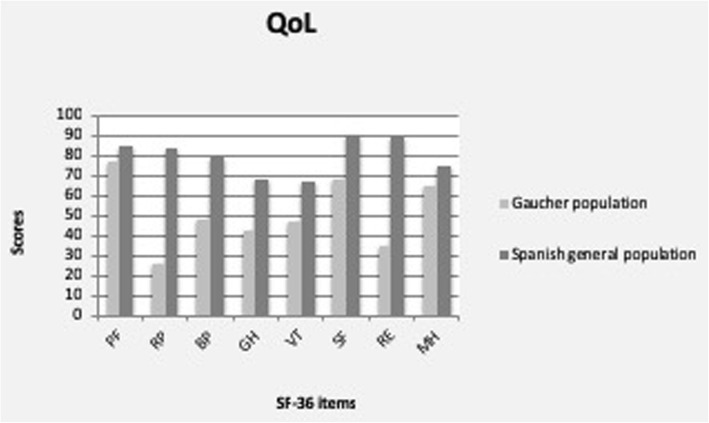
SF36 questionnaire. Score distribution between GD patients and Spanish general population

Figure 2 shows the graphic analysis of the QoL status of the GD patients compared to the normal population values of reference.

## Discussion

Chronic fatigue is the most prevalent symptom in patients with Gaucher disease; it appears at any age and usually the patients are not aware of the symptom/sensation, until they perceive the improvement after starting a specific treatment for the disease (either enzymatic replacement therapy or substrate reduction therapy). In some studies, no association was found between enzymatic replacement therapy and the QoL and it is recognized in the surveys made by physicians treating patients with Gaucher disease that fatigue is the most persistent symptom [2].

There are few reports about the significance and origination of this symptom. Many interpretations and theories have been taken into account, regarding the role of different factors such as the altered glyco-lipid metabolism, anemia, bone disease, and comorbidities but there is not a clear explanation yet [1, 2].

Bone affectation is today a well-known and growing area of research on Gaucher disease. There are studies of bone disease pathophysiology, mechanisms of bone infiltration and microenviroment changes, and vascular intraosseous events; decreased bone density has also been extensively examined [1, 4, 5]. Nevertheless, the muscle-tendon function has been little explored in patients with Gaucher disease.

Real-time tissue elastography is a new tool that helps to estimate the internal tissue strain by means of ultrasound radiofrequency. The method is based on the compression of external tissue, with subsequent computation of the strain profile along the transducer axis, which is derived from the cross-correlation analysis of pre- and post-compression. The strain profile can then be converted into an elastic modulus profile by measuring the stress applied by the compressing device and applying certain corrections for the non-uniform stress field. These data can then be translated into color images indicating the degree of elasticity of the tissue.

In short, it is a non-invasive technique that allows the identification of a region of the tissue with a different elasticity than the surrounding areas. This is achieved by simultaneously strain measuring and stress patterns in the tissue portion using an ultrasonic imaging system combined with a pressure-sensing array [9, 10].

The new applications of the elastography provide a low cost non-invasive and not ionization technology as a research tool to assess the mechanical properties of musculotendinous tissues and detect subclinical changes of muscles and tendons. It could potentially be valuable for early diagnosis and for monitoring during rehabilitation medicine [17–20]).

The Achilles tendon is the most powerful and thick tendon of the musculoskeletal system, but it is the one that most often suffers traumatisms. Up to 50% of athletes can suffer damage in the Achilles tendon to its structure [17, 18]. Also, metabolic chronic diseases such as diabetes can modify the structure and biomechanical properties of the Achilles tendon producing a degenerative tendinopathy. Diabetes produces an architectural disorganization of the tendinous fibers; however, the neuropathy that induces diabetes could justify a decrease in the mobility of the extremities and as a consequence a decrease in tendon stiffness [21, 22].

The structure of the bones, cartilage, and tendons is constituted by collagen fibers, especially in the tendons. As collagen is produced by the fibroblasts and these cells are directly compromised by the lysosomal accumulation of glucosylceramide. It could be thought about a subclinical dysfunction of this tissue secondary to substrate accumulation. Although the purpose of our study was to assess the tendon elasticity, the simultaneous assessment in B mode excluded the existence of glycolipid accumulations that were sonographically detectable.

It is important to consider the role of ionic channels in tissue functioning; it is known that in sphingolipidosis in general, there are defects in the traffic of lipids in the endosome, lysosome, and autophagosomes with accumulation of lipids inside the lysosomes which produces defects in membrane traffic and alteration in homeostasis of Ca_2_^+^ [22]. Our hypothesis that tendon dysfunction could be justified in some patients by a glycol-lipid overload in fibroblasts would link with the evidence reflected in the study conducted by our group that shows evidence for such defective KCa3.1 regulation in Fabry disease fibroblasts and in Niemann-Pick C fibroblasts, disturbances in membrane trafficking, or function in the fibroblasts [23]. As well as in another study on KCa3.1 functions performed in type-1 and -3 GD patients, we found a defect in the physiologically occurring upregulation of KCa3.1 during monocyte-to-macrophage differentiation [24, 25], suggesting that KCa3.1 dysregulation is a general feature of sphingolipidosis and cellular pathophysiology.

In the present study, no significant correlation was observed between the tendinous anomaly detected by elastography and the levels of the biomarkers; however, it is noteworthy that over 70% of the studied patients, after many years on therapy, continued showing an increased level of biomarkers. These persistent high levels can be assumed as indirect indicators of substrate accumulation and macrophage hyperactivity; in consequence, these annotations reinforce their theoretical relationship with the symptomatic fatigue.

It is important to remark that in our study there is no age or gender bias; all patients have a long-term exposition to therapy, and they do not have anemia or iron deficiency; however, the persistence of high biomarkers stands out.

The tendon alteration is not related to the intensity of the bone disease or the weakness of the mineral density; also, no relationships with genotype, spleen removal, time on therapy, or comorbidities were found. A possible interpretation of the findings obtained in this study suggests that mechanical overload on lower limbs is the cause of tendon dysfunction.

We propose that exploring tendon elasticity produces useful information in the evaluation of chronic fatigue presented by patients with GD. The detection of subclinical alterations in the Achilles tendon is valuable information to prevent musculoskeletal complications.

The identification of tendon stiffness would be a sign of alarm, focused on the risk of bone complications such as fracture or degenerative arthropathy, and oriented toward the opportunity to propose a program of physical therapeutic exercise as prophylaxis of musculoskeletal complications. Also, strain-elastography could be an interesting and useful tool to monitoring the evolution and response to the physical therapy. It could provide insights for physical medicine and rehabilitation researchers respect to tendon properties and their impact on functionality.

### Limitations

As in the majority of GD papers, one of the most severe limitations is the number of patients. The small sample can justify the absence of statistically significant results, which can appear if the number of analyzed patients was higher.

Certain aspects of strain-elastography are controversial, such as its dependence on a well-trained operator, and there can be variability between different operators with impact in the reproducibility of the obtained results. In our study, in order to minimize the variability, all evaluations were carried out with the same device and technique and interpreted by the same expert specialist; this permitted a reliable way to integrate the information collected from the evaluation of bone marrow MRI findings, ultrasonography densitometry, and form the strain-elastography.

This is the first study with strain-elastography performed in Achilles tendon in a group of GD patients.

## Conclusions

Strain-elastography is a low-cost sensitive, painless, and fast method to detect tissue alterations. Strain-elastography in Achilles tendon is a conscious method to detect subclinical dysfunction of muscle-tendinous unit that could contribute to explain partially the fatigue symptom in GD.

Intratendinous alteration detected by the strain-elastography is an independent variable in GD patients in this study. The cause of this dysfunction could be probably a mechanical cause related to overload on lower limbs. In consequence, this fact can be a risk factor for the development of skeletal complications.

We propose that exploring tendon elasticity produces useful information in the evaluation of chronic fatigue presented by patients with GD. The detection of subclinical alterations in the Achilles tendon is valuable information to prevent musculoskeletal complications.

A project based on physical activity has been developed in GD patients to increase the functional strength of the lower limbs to achieve greater stability, power, and improvement in physical activities, as well as the reduction of fatigue.

## Supplementary information

**Additional file 1:** **Table S1.** General characteristics.

## Data Availability

There are available.

## Abbreviations

BMD: Bone mineral density
BUA: Broad ultrasound attenuation
CCL18/PARC: `Chemokine (C-C motif) ligand 18/pulmonary and activation-regulated chemokine
CEICA: Ethical Committee of Aragon Research Community
CFg: Chronic fatigue
ChT: Chitotriosidase activity
ERT: Enzymatic replacement therapy
GD: Gaucher disease
GD1: Type 1 Gaucher disease
GD2: Type 2 Gaucher disease
GD3: Type 3 Gaucher disease
Lyso Gb1: Plasma glucosylsphingosine
MRI: Magnetic resonance imaging
QoL: Quality of life
S-ELA: Strain-elastography
SF-36: Short Form questionnaire
S-MRI: Spanish MRI score
SPSS: Statistical Package for the Social Sciences
UPLC: Liquid chromatography gradient
