# Supplementary material for: Muscle-tendon weakness contributes to chronic fatigue syndrome in Gaucher’s disease
Source: J Orthop Surg Res. 2019 Nov 21;14:383. doi: 10.1186/s13018-019-1452-y (PMC6873505; doi:10.1186/s13018-019-1452-y)
Supplement: Supplementary file 1 — Additional file 1: Table S1. General characteristics. [file 13018_2019_1452_MOESM1_ESM.docx]

General characteristics (Supplementary material)

| Patient | M/F | Age (y) | Time on T  herapy (y) | Type of therapy | Genotype  (NM_000157)  (NP_000148) | Liver volumen  by MRI (mL) | Spleen volumen  by MRI (mL) | S-MRI | T score | Comorbiliities |
| --- | --- | --- | --- | --- | --- | --- | --- | --- | --- | --- |
| 01 | F | 43 | 25 | Imi/Vela/Eliglustat | [c.108G>A]+[c.1226A>G]  [p.Trp36Ter]+[p.Asn409Ser] | 1048 | 170 | 12 | -0.24 | Bone crisis. Joint eplacement.  Ca Uterus |
| 02 | M | 40 | 7 | Velaglucerase | [c.84dupG]+[c.1226A>G]  [p.Leu29AlafsTer18]+[p.Asn409Ser] | 1223 | 248 | 2 | 1.51 | NO |
| 03 | M | 59 | 20 | Imiglucerase | [c.1226A>G]+[c.1448T>C]  [p.Asn409Ser]+[p.Leu483Pro] | 1311 | 370 | 5 | -0.77 | HTA |
| 04 | M | 55 | 2 | Velaglucerase | [c.371A>G]+[c.1448T>C]  [p.Tyr244Cys]+[p.Leu483Pro] | 1828 | 955 | 9 | -1.03 | Epilepsy by traumatism |
| 05 | M | 50 | 3 | Imiglucerase | [c.371A>G]+[c.1448T>C]  [p.Tyr244Cys]+[p.Leu483Pro] | 1450 | - | 11 | -0.55 | Splenectomy. Joint replacemant  Tachicardia |
| 06 | F | 58 | 9 | Miglustat | [c.1226A>G]+[c.1448T>C]  [p.Asn409Ser]+[p.Leu483Pro] | 1082 | 260 | 2 | -1.42 | NO |
| 07 | M | 53 | 18 | Imi/Velaglucerase | [c.1226A>G]+[c.1448T>C]  [p.Asn409Ser]+[p.Leu483Pro] | 1287 | 333 | 0 | 1.23 | Vertebral discopathy |
| 08 | M | 18 | 13 | Imi/Velaglucerase | [c.1448T>C]+[c.1448T>C]  [p.Leu483Pro]+[p.Leu483Pro] | 1014 | 105 | 0 | -1.78 | Kyphoscoliosis |
| 09 | F | 49 | 2 | Velaglucerase | [c.1226A>G]+[c.1448T>C]  [p.Asn409Ser]+[p.Leu483Pro] | 1296 | 93 | 15 | -2.09 | Muscle atrophies. Glaucoma |
| 10 | F | 22 | 18 | Imi/Velaglucerase | [c.84dupG]+[c.1226A>G]  [p.Leu29AlafsTer18]+[p.Asn409Ser] | 708 | 219 | 6 | -2.3 | Bone crisis |
| 11 | F | 65 | 25 | Imi/Tali/Vela | [c.577A>C]+[c.1226A>G]  [p.Thr193Pro]+[p.Asn409Ser] | 870 | - | 6 | -2.0 | Splenectomy. Fractures.  Ca colon |
| 12 | M | 55 | 19 | Imi/Tali/Vela | [c.1124T>C]+[c.1226A>G]  [p.Leu375Pro]+[p.Asn409Ser] | 890 | - | 8 | 0.72 | Splenectomy. Multiple Joint  replacement |
| 13 | F | 56 | 20 | Miglustat | [c.721G>A]+[c.1226A>G]  [p.Gly241Arg]+[p.Asn409Ser] | 902 | 140 | 4 | -2.68 | Raynaud. Bladder Ca |
| 14 | M | 58 | 10 | Vela/Eliglustat | [c.1226A>G]+[c.1207Ins]  [p.Asn409Ser] | 880 | - | 18 | -2.73 | Splenectony. Degenerative  polyarthriopathy |
| 15 | F | 54 | 20 | Imi/Velaglucerase | [c.1226A>G]+[c.1448T>C]  [p.Asn409Ser]+[p.Leu483Pro] | 1200 | - | 8 | -2.20 | Splenectomy. Meningioma |
| 16 | M | 47 | 18 | Miglustat | [c.1226A>G]+[c.1448T>C]  [p.Asn409Ser]+[p.Leu483Pro] | 1420 | - | 8 | 1.12 | Splenectomy |
| 17 | F | 51 | 15 | Miglustat | [c.1226A>G]+[c.1448T>C]  [p.Asn409Ser]+[p.Leu483Pro] | 925 | 301 | 4 | -1.49 | Carpal tunnel |
| 18 | F | 20 | 2 | Velaglucerase | [c.1226A>G]+[c.1448T>C]  [p.Asn409Ser]+[p.Leu483Pro] | 1999 | 1358 | 11 | -0.48 | NO |
| 19 | F | 23 | 18 | Imi/Tali/Vela | [c.1226A>G]+[c.1448T>C]  [p.Asn409Ser]+[p.Leu483Pro] | 1050 | 170 | 0 | -0.98 | NO |
| 20 | M | 22 | 21 | Imi/Velaglucerase | [c.1246G>A]+[c.1504C>T]  [p.Gly416Ser]+[p.Arg502Cys] | 878 | 230 | 0 | 0.37 | NO |
| 21 | M | 22 | 10 | Imiglucerase | [c.1226A>G]+[c.1448T>C]  [p.Asn409Ser]+[p.Leu483Pro] | 1150 | 383 | 7 | 1.27 | NO |
| 22 | F | 30 | 14 | Imiglucerase | [c.1226A>G]+[c.1448T>C]  [p.Asn409Ser]+[p.Leu483Pro] | 1198 | 232 | 12 | -2.29 | Neutrophilic dermatitis |
| 23 | F | 19 | 16 | Imiglucerase | [c.256C>T]+[c.1226A>G]  [p.Arg86Ter]+[p.Asn409Ser] | 1050 | 222 | 8 | 0.56 | NO |
| 24 | M | 21 | 3 | Velaglucerase | [c.517A>C]+[c.1226A>G]  [p.Thr173Pro]+[p.Asn409Ser] | 1764 | 627 | 12 | -2.34 | NO |
| 25 | M | 62 | 8 | Velaglucerase | [c.1226A>G]+[c.1448T>C]  [p.Asn409Ser]+[p.Leu483Pro] | 1157 | 657 | 7 | 1.11 | Eczema |
| 26 | M | 40 | 10 | Miglustat | [c.1226A>G]+[c.1448T>C]  [p.Asn409Ser]+[p.Leu483Pro] | 1798 | 427 | 13 | 1.2 | Vertebral discopathy |
| 27 | M | 18 | 7 | Velaglucerase | [c.1226A>G]+[R130W]  [p.Asn409Ser] | 1016 | 375 | 4 | -0.68 | NO |
| Total | **M:15**  **F:12** | **41.0**  **18-62** |  |  |  | **1207**  **708-1999** | **375**  **93-1358** | **7.1**  **0-18** | **-0.67**  **1.51;-2.39** |  |

M: male; F: female. Imi: Imiglucerase; Vela: Velaglucerase alfa; Tali: Taliglucerase alfa; S-MRI: Spanish MRI score

**Figure legends**

Figure 1. Strain-Elastography of Achilles tendon. A: Sagital view. Normal stiffness (blue color). B: Sagittal view. Abnormal elasticity (red color). Fibrilar structure preserved.

Figure 2. SF36 questionnaire. Score distribution between GD patients and Spanish general population
